# Supplementary material for: Synergistic Interactions between Tocol and Phenolic Extracts from Different Tree Nut Species against Human Cancer Cell Lines
Source: Molecules. 2022 May 14;27(10):3154. doi: 10.3390/molecules27103154 (PMC9143696; doi:10.3390/molecules27103154)
Supplement: Supplementary file 1 [file molecules-27-03154-s001.zip › molecules-1700913-supplementary.pdf]

Table S1. Effect of TRE and PCE on the viability of cancer cells

Nut	HeLa	MCF7	A549	PC3	ARPE
TRE (800 µg/mL)					
ALM	56.0±1.7^{b}	84.6±7.1^{c}	101.7±5.6^{d}	91.9±9.4^{d}	102.0±5.4^{a}
EOA	47.1±2.5^{a}	66.6±10.2^{a}	81.1±5.9^{b}	70.5±8.9^{ab}	99.7±6.1^{a}
WN	53.5±4.4^{ab}	70.2±6.9^{ab}	88.0±4.4^{bc}	63.3±9.8^{a*}	101.8±4.9^{a}
PN	56.8±6.7^{b}	77.9±8.0^{abc}	88.72±3.3^{c}	80.3±4.2^{bcd}	101.18±4.6^{a}
WPN	56.3±2.7^{b}	75.3±2.7^{abc}	65.82±6.4^{a}	74.5±11^{abc}	101.0±1.9^{a}
PPN	56.7±3.2^{b}	71.1±10.5^{ab}	82.19±6.2^{bc}	64.4±12^{a*}	100.9±3.1^{a}
PT	55.7±7.7^{b}	81.5±7.3^{bc}	104.1±2.4^{d}	87.3±4.4^{cd}	101.0±2.0^{a}
PRE (800 µg/mL)					
Nut	HeLa	MCF7	A549	PC3	ARPE
ALM	93.9±4.6^{a}	83.5±5.4^{ab}	99.29±2.5^{c}	110.7±7.3^{c}	135.60±7.2^{b}
EOA	95.1±7.4^{a}	81.8±7.3^{ab}	87.7±3.3^{ab}	91.2±5.6^{ab}	132.4±8.0^{b}
WN	85.0±1.7^{a}	80.3±13.8^{ab}	83.0±5.2^{a}	85.7±4.6^{a}	147.55±4.0^{c}
PN	86.6±4.6^{a}	75.2±6.0^{a}	88.69±4.8^{b}	93.5±5.2^{b}	157.24±8.1^{c}
WPN	101.0±7.0^{a}	86.3±4.4^{ab}	89.31±2.7^{b}	95.9±3.6^{b}	128.4±8.0^{ab}
PPN	95.3±2.6^{a}	86.0±10.3^{ab}	100.8±3.1^{c}	92.3±7.2^{b}	130.9±4.5^{ab}
PT	89.7±4.2^{a}	92.3±8.1^{b}	97.5±2.0^{c}	94.1±7.6^{b}	120.9±6.7^{a}

Different superscript letters in the same column indicate significant difference between extracts on the same cell line

Table S2. Selectivity of the cytotoxicity of tocol and phenol extracts in cancer cell lines

TRE	HeLa	MCF7	A549	PC3	ARPE
Alm	1.75	1.16	0.95	1.07	1
EOA	2.33	1.65	1.35	1.56	1
WN	1.69	1.42	1.14	1.58	1
PN	1.90	1.39	1.22	1.35	1
WPN	1.80	1.34	1.54	1.32	1
PPN	2.34	1.85	1.60	2.04	1
PT	1.66	1.14	0.89	1.06	1
Average	1.92	1.42	1.24	1.42	1
PCE	HeLa	MCF7	A549	PC3	ARPE
Alm	1.23	1.38	1.16	1.04	1
EOA	1.22	1.40	1.32	1.27	1
WN	1.16	1.24	1.18	1.16	1
PN	1.33	1.56	1.30	1.23	1
WPN	1.29	1.51	1.46	1.36	1
PPN	1.22	1.34	1.15	1.25	1
PT	1.13	1.09	1.03	1.07	1
Average	1.23	1.36	1.23	1.20	1

Table S3. EC20 and EC50 of extracts alone and in combination

	TRE		PRE		EC50 COMBINATION	
	EC20 µg	EC50 µg	EC20 µg	EC50 µg	EC20 PRE: TRE	EC20 TRE: PRE
HeLa						
EOA	244.98 ± 17.56	950.99 ± 71.75	1671.36 ± 10.28	7856.49 ± 198.65	302.51 ± 0.78	82.07 ± 1.72
PN	276.10 ± 2.42	1047.18 ± 5.75	1402.35 ± 26.67	8316.10 ± 194.01	283.68 ± 0.10	151.96 ± 2.66
WN	214.22 ± 16.84	951.4 ± 32.16	1498.41 ± 89.45	6078.80 ± 402.31	259.86 ± 0.04	220.80 ± 1.48
MCF7						
EOA	482.18 ± 8.80	2092.3 ± 439.41	879.02 ± 295.95	2847.87 ± 145.56	500.60 ± 24.50	208.68 ± 30.85
PN	984.80 ± 207.56	9040.81 ± 458.94	657.77 ± 24.44	2037.75 ± 270.82	441.76 ± 21.67	353.07 ± 11.14
WN	522.30 ± 49.90	2523.8 ± 418.17	711.25 ± 235.96	2103.80 ± 624.17	242.00 ± 5.44	34.19 ± 3.07

EC20 and EC50 from Tocopherol rich extract (TRE) and phenolic rich extract (PRE) alone and in combination (Mean ± SEM). Each EC50 and EC20 was calculated using a linear equation by log (dose) response curves.
